# Protocol for measuring mitochondrial respiration in mature human adipocytes using the Seahorse XF analyzer

**DOI:** 10.1016/j.xpro.2026.104520

**Published:** 2026-04-25

**Authors:** Helen Broghammer, Claudia Gebhardt, John T. Heiker

**Affiliations:** 1 Helmholtz Institute for Metabolic, Obesity and Vascular Research (HI-MAG) of the Helmholtz Zentrum München at the University of Leipzig and University Hospital Leipzig, Leipzig, Germany

**Keywords:** Cell Biology, Cell culture, Cell isolation, Metabolism

## Abstract

Primary mature human adipocytes are an important tool bridging basic research and clinical medicine by reflecting human biological diversity. Here, we present a protocol for measuring the oxygen consumption rate of mature adipocytes. We describe the steps for isolation of adipocytes from adipose tissue samples, Matrigel embedding, and Seahorse analysis, as well as normalization and data analysis. This protocol enables the investigation of the metabolic function of primary mature human adipocytes in response to drugs or genetic modifications.

## Highlights

• Steps for the isolation and handling of viable primary human mature adipocytes
• Instructions for embedding of floating adipocytes in Matrigel for Seahorse analysis
• Guidance on Seahorse XF setup for OCR and ECAR measurements of mature adipocytes
• Procedure for normalizing OCR measurements tailored for primary mature adipocytes

## Abstract (editor highlights)

Publisher’s note: Undertaking any experimental protocol requires adherence to local institutional guidelines for laboratory safety and ethics.

## Graphical abstract

**Figure:**
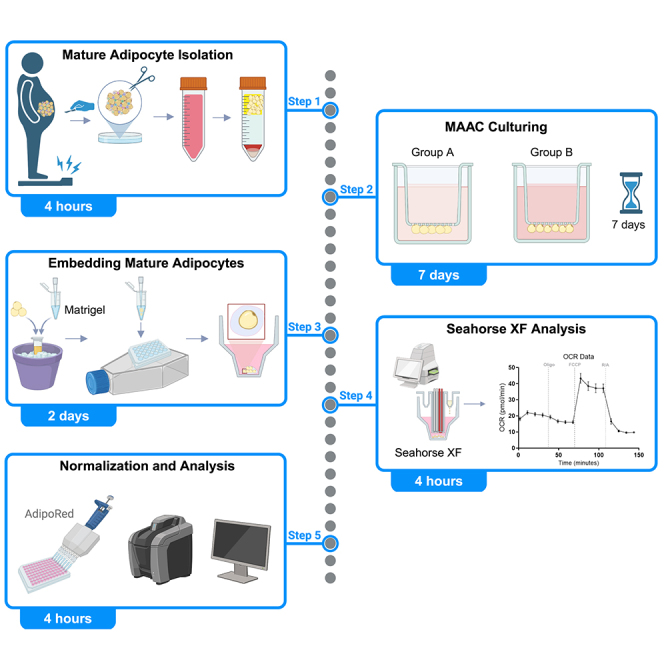

## Before you begin

The Seahorse extracellular flux technology enables the real-time measurement of mitochondrial respiration in adherent cell types and tissue explants. However, applying this technology freshly isolated mature adipocytes has been technically challenging, as they are difficult to attach securely to the bottom of the well due to their high lipid content and buoyancy. Consequently, pioneering work and protocols have relied on stromal vascular fraction (SVF)-derived adipocytes that are differentiated in vitro, or on bulk adipose tissue/explant preparations that contain multiple non-adipocyte cell types.^1^ An oxygraph system has been used to perform high-resolution respirometry on human mature adipocytes,^2^ allowing a detailed evaluation of mitochondrial respiratory capacity under specific substrate conditions. However, it does not provide the multiplexed, higher-throughput measurements possible with the Seahorse platform, such as simultaneous measurements of oxygen consumption rate (OCR) and extracellular acidification rate (ECAR).

### Innovation

The key innovation of this protocol is that it allows Seahorse XF metabolic analysis to be applied to freshly isolated, in vivo-differentiated, primary, human mature adipocytes. Based on recent advances in membrane mature adipocyte aggregate cultures (MAAC),^3^ we developed a Matrigel-based embedding strategy that allows for the direct and robust measurement of OCR and ECAR, providing broader bioenergetic profiling and greater experimental throughput. Expanding on earlier pioneering work,^1^^,^^2^ the protocol integrates optimized procedures for adipocyte isolation, Matrigel embedding, and assay medium formulation, including Seahorse analyzer configuration and normalization strategies tailored to mature adipocytes. This workflow enables the direct assessment of mitochondrial respiration in a physiologically relevant human model, offering a valuable tool for investigating adipocyte function and dysfunction in obesity and metabolic disease.

### Institutional permissions

#### Study approval: Leipzig Obesity BioBank

Written informed consent was obtained from all patients. All studies were approved by the Ethics Committee of the University of Leipzig (approval numbers: 159-12-21052012 and 017-12ek) and performed in accordance with the Declaration of Helsinki, the Bioethics Convention (Oviedo), and EU Directive on Clinical Trials (Directive 2001/20/EC). All AT donors have been informed of the purpose, risks and benefits of the biobank. Ethical guidelines and EU legislation for privacy and confidentiality in personal data collection and processing is being followed, directive 95/46/EC.

For all studies using human tissues, permission from the relevant institutions need to be acquired.

### Prepare reagents and materials

**Timing: 1 week prior to the assay**

1. Prepare dissociation buffer, wash buffer and CM as described in the “materials and equipment” section.
2. Sterilize scissors, forceps, spatulas and tea strainer used for the preparation of the tissue.

## Key resources table

**Table:** 

REAGENT or RESOURCE	SOURCE	IDENTIFIER
**Biological samples**		

Adipose tissue	Plastic Surgery	N/A

**Chemicals, peptides, and recombinant proteins**		

Bovine Serum Albumin (BSA)	Carl Roth GmbH	8076.2
CaCl_2_	Carl Roth GmbH	CN93.1
Collagenase type II	Gibco	17101015
DMEM/F12	Gibco	31330038
Fetal calf serum (FCS)	Sigma-Aldrich	S0615
Glucose	Carl Roth GmbH	HN06.2
HEPES	Carl Roth GmbH	9105.4
KCl	Carl Roth GmbH	6781.3
Krebs-Henseleit Buffer	Sigma-Aldrich	K3753
NaCl	Carl Roth GmbH	3957.2
PBS	Gibco	10010023
NaHCO_3_	Carl Roth GmbH	0965.2
ZellShield®	Minerva Biolabs	13–0050
Corning® Matrigel® Basement Membrane Matrix	Sigma-Aldrich	CLS356237-1EA
Seahorse XF DMEM, pH 7.4	Agilent	103680–100
Seahorse XF 1.0 M glucose solution	Agilent	103577–100
Seahorse XF 100 mM pyruvate solution	Agilent	103578–100
Seahorse XF 200 mM glutamine solution	Agilent	103579–100

**Software and algorithms**		

Wave Desktop and Controller	Agilent	
Ilastik	Berg et al.^4^	https://doi.org/10.1038/s41592-019-0582-9
CellProfiler™	Stirling et al.^5^	https://doi.org/10.1186/s12859-021-04344-9

**Other**		

20 ml syringe	BD®	302237
0.4 μm cell culture plate Inserts	VWR®	734–2720
Forceps	Carl Roth GmbH	2687.1
Scissors	Carl Roth GmbH	3545.1
Spatula	Carl Roth GmbH	K749.1
Stainless steel tea strainer	N/A	N/A
21 gauge blunt end cannula	B. Braun	4665643
300 μm syringe Strainer	pluriSelect®	43-71300-50
6-well cell culture plate	SARSTEDT	83.3920.005
Seahorse XF Pro M cell culture microplate	Agilent	103775–100
Seahorse XFe96 Pro Extracellular Flux Assay Kit	Agilent	103775–100
Seahorse XF Calibrant Solution	Agilent	103775–100
Seahorse XF Cell Mito Stress Test Kit	Agilent	103015–100
Seahorse XF Pro Analyzer	Agilent	
BX-Z800 Microscope	Keyence	

## Materials and equipment

**Table:** Dissociation Buffer

Reagent	Final concentration	Amount
NaCl	123 mM	7.2 g
KCl	5 mM	372.8 mg
CaCl_2_	1.3 mM	144.3 mg
Glucose	5 mM	900.8 mg
HEPES	100 mM	23.8 g
ZellShield®	1%	10 mL
BSA	4%	40 g
distilled water	N/A	990 mL
**Total**	**N/A**	**1000 mL**

Sterilize by filtering through a 0.22 μm filter system, aliquot and store at −20°C.

***∗Ready-to-use dissociation buffer:*** Add 0.2% collagenase type II shortly before use.

**Table:** Wash Buffer

Reagent	Final concentration	Amount
Krebs-Henseleit Buffer	N/A	1 vial
CaCl_2_	2.5 mM	277.5 mg
NaHCO_3_	25 mM	2.1 g
HEPES	25 mM	5.9 g
BSA	1%	10 g
distilled water	N/A	up to 1000 mL
**Total**	**N/A**	**1000 mL**

Adjust the pH to 7.2. Sterilize by filtering through a 0.22 μm filter system, aliquot and store at −20°C.

***Note:*** Before adding it to the solution, resuspend CaCl_2_ in a small volume of distilled water.

**Table:** Cultivation Media (CM)

Reagent	Final concentration	Amount
DMEM/F12	N/A	445 mL
Fetal calf serum (FCS)	10%	50 mL
ZellShield®	1%	5 mL
**Total**	**N/A**	**500 mL**

CM that has been prepared can be stored at 4°C for up to one week.

### Human subcutaneous adipose tissue collection

SAT samples were collected during elective aesthetic and postbariatric surgery at the Division of Plastic, Aesthetic and Special Hand Surgery of University Hospital Leipzig. All operations were performed under general anesthesia. Patients with prior liposuction or cryolipolysis to the respective area were excluded from sample collection. Electrocautery was used to prepare subcutaneous tissue for resection. Thermally damaged tissue and skin were removed using scissors or scalpel, and fat samples were placed into sterile sample containers for immediate processing.

***Note:*** To obtain enough floating, mature adipocytes for a Seahorse XF 96-well plate, we recommend isolating them from 5–10 g of human adipose tissue, depending on whether the tissue is from patients without or with obesity. The stability, viability and ultimately number of adipocytes obtained does vary widely between patients, and, in our experience, may depend on age and BMI.

## Step-by-step method details

### Isolation of mature human adipocytes

The protocol below outlines the steps for isolating and cultivating mature human adipocytes as membrane adipocyte aggregate cultures (MAAC,^3^). To obtain a high number of mature adipocytes from tissue samples, it is crucial to quickly process the adipose tissue. Avoid storing adipose tissue in a refrigerator or on ice prior to the isolation process, as this will dramatically reduce the number of living mature adipocytes. All tissue handling steps are carried out under a sterile workbench.

Duration: 4–5 h.

1. Add 0.2% collagenase type II to the prepared dissociation buffer. Prewarm the buffer to 37°C in a water bath.
2. Hold the adipose tissue firmly with a forceps and use a spoon-shaped spatula to scrape the adipose tissue away from the surrounding connective tissue.
3. Use scissors to cut the adipose tissue into smaller pieces, approximately 1–3 mm^3^.
4. Place the adipose tissue in a 50 ml tube and add the prewarmed dissociation buffer to achieve a tissue-to-buffer ratio of approximately 1.5:1 (e.g., 30 mL of tissue and 20 mL of dissociation buffer).
5. Close the tube tightly and place it in a prewarmed water bath at 37°C.
6. Incubate for 30–45 min, carefully inverting the tube every 5–10 min.

***Note:*** Prolonged incubation (more than 50 min) may compromise adipocyte viability.

7. Remove the tubes from the water bath and filter through a coarse filter to remove larger debris.

***Note:*** For this filtration step, we use a heat-sterilized stainless steel tea strainer.

8. Attach a 21-gauge blunt end cannula to a 20 mL syringe.
9. Draw the adipocyte solution up into a 20 mL syringe and filter it through a 300 μm syringe Strainer.
10. Centrifuge the tube at 100 × *g* at 20°C–25°C for 2 min.
11. The adipocytes will now float on top. Insert a syringe needle into the 50 mL tube and remove the infranatant which contains the dissociation buffer and stromal vascular fraction.
12. Add 10 mL of wash buffer. Invert the tube gently 8–10 times. Centrifuge at 100 × *g* at 20°C–25°C for 2 min.
13. Remove the infranatant and repeat the washing step until a densely packed adipocyte fraction is achieved.

***Note:*** At the beginning of the washing process, two fractions are visible after centrifugation: the infranatant with buffer, cell debris and stromal vascular fraction and the supernatant containing the mature adipocytes. As you repeat the washing steps, a third fraction will appear on top of the adipocyte fraction. This fraction contains free lipids from collapsed adipocytes. Remove this fraction as well. To obtain good results, it is crucial to work with pure, mature adipocytes that are free of buffer and extracellular lipids.

**Figure 1:**
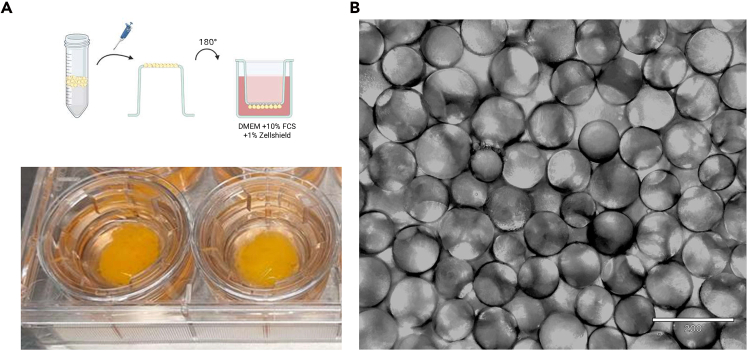
Cultivation of mature adipocyte aggregate cultures (MAAC) (A) Primary mature human adipocytes cultured under transwells as MAACs. Created in BioRender (https://BioRender.com/sg0ws3e). (B) A representative image of MAACs with a 10 × magnification; scale bar: 200 μm.
17. Add 2 mL of CM to the inner well.
18. Place the lid on the plate and carefully transfer it to a cell culture incubator.
19. Incubate in a humidified incubator at 37°C with 5% CO_2_.
20. For experiments, where a different or supplemented CM is used, perform a medium exchange every other day. Be careful not to aspirate adipocytes during the process.

***Note:*** To demonstrate an effect of culturing conditions on adipocyte mitochondrial respiration in the exemplary OCR measurements, group A was cultured with regular CM, and group B was cultured with supplemented CM for 7 days.

***Note:*** Mature adipocytes can be cultured in MAACs for up to 14 days while maintaining viability, as well as adipocyte- and depot-specific gene expression, without dedifferentiation.^1^ OCR measurements can also be performed earlier; however, we recommend waiting at least one day after isolation before performing Matrigel embedding to allow the adipocytes to recover from stress induced by the isolation process.

14. Once you have achieved a densely packed adipocyte fraction that is almost free of buffer and free lipids, let it rest at 20°C–25°C for ∼15 min.
  a. In the meantime, prepare the 6-well cell culture plate and the 0.4 μm cell culture plate Inserts (transwells).
  b. Pipette 4 mL of CM into each well of a 6-well cell culture plate.
  c. Remove the transwells from the package.
  d. Place them upside down on the sterile workbench.
  e. Use a gloved thumb to make a small indentation in the membrane.
15. Pipette 300–750 μL of adipocytes onto each membrane.
16. Quickly invert the transwell and place it in one of the prepared wells immediately (Figure 1).

### Embedding of mature human adipocytes into Matrigel

**Timing: 2 days prior to the Seahorse assay**

The following section provides detailed instructions for the embedding of mature adipocytes in Corning® Matrigel® Basement Membrane Matrix.

21. Thaw the Matrigel on ice. Precool the wide-bore pipette tips and combi tips at −20°C.

***Note:*** Matrigel is available in different formulations (e.g., standard or growth factor–reduced), and users should select the appropriate product type according to their specific experimental requirements. Due to limited tissue availability and restricted culture duration, we did not systematically evaluate potential lot-to-lot effects of Matrigel, which should therefore be considered when interpreting results. However, subtle lot-specific differences may be masked by the inherent biological variability between donors.

22. Harvest adipocytes cultivated as MAACs.
  a. Prepare a 5 mL tube with PBS.
  b. Remove the plate from the incubator and aspirate the cell culture medium.
  c. Remove the transwell and carefully rinse the cells from the transwell into the well.
  d. Collect the cells in the PBS and transfer the adipocytes to the prepared 5 mL tube with PBS.

***Note:*** To minimize mechanical stress on the cells, make sure to use wide-bore pipette tips and slowly collect the adipocytes.

23. Gently invert the tube 8–10 times. Then, centrifuge at 100 × *g* at 20°C–25°C for 2 min to separate the adipocytes from the lipids.
24. Insert a syringe needle and remove the infranatant.

***Note:*** If necessary, also remove any free lipid layer from the top of the adipocytes using a 10 μL pipette. Repeat this step until all of the free lipids are removed.

**CRITICAL:** Accurate removal of the free lipid layer is crucial at this stage. Otherwise, it will interfere with Matrigel polymerization.

25. Place the 5 mL tubes containing the cells on ice for 1 min.
26. Fill a T175 flask with hot water and place the Seahorse 96XF cell culture plate on top.
27. Prepare a cell-Matrigel-mixture.
  a. Mix Matrigel with the cells at a ratio of 1:1 using a precooled wide-bore pipette tip.
  b. Pipet carefully up and up and down a few times.
  c. Immediately transfer 10 μl of the cell suspension into each well of the pre-warmed Agilent Seahorse XF Pro M cell culture microplate using a multistep pipette with pre-cooled combi tip.
  d. Distribute the cells by tapping the plate vertically and place it into the incubator.

**CRITICAL:** Work quickly, as small volumes of Matrigel polymerize rapidly at 20°C–25°C. Set the draw-up and dispensing speed of the combi-tip pipette to a maximum of 4 to minimize mechanical stress on the cells.

28. After the Matrigel has completely polymerized, which takes approximately 2–3 min, fill the wells with cell CM.

***Note:*** This process leads to cell stress; therefore, always embed the cells 1–2 days prior to the assay. Some cells may detach during the assay or not survive the embedding process.

### Cartridge hydration

**Timing: 1 day prior to the Seahorse assay**

Here, we describe the hydration process for the Seahorse XFe96 Pro Extracellular Flux Assay Kit sensor cartridge. It is critical to use a fully hydrated Seahorse cartridge for the following analysis; therefore we recommend hydrating the cartridge for at least 16 h.

29. Start the Seahorse XF Pro Analyzer, then connect to the Wave desktop and controller software.

***Note:*** Allow Seahorse XF Analyzer to heat up for a minimum of 16 h.

30. Hydrate the sensor cartridge.
  a. Place the cartridge upside down, next to the utility plate.
  b. Fill each well of the utility plate with 200 μL of Seahorse XF Calibrant Solution.
  c. Place the XF Hydrobooster on top of the utility plate and push down to ensure a tight seal.
31. Incubate the sensor cartridge for at least 16 h at 37°C in a CO_2_-free incubator.

### XF cell mitochondrial stress test

**Timing: 1 h prior to the assay**

This section provides detailed instructions for a Cell Mito Stress Test (MST) with optimized conditions for OCR measurements in mature adipocytes embedded in Matrigel.

32. Prepare the Seahorse assay medium (AM) as stated in Table 1 and place at 37°C in a water bath.

**Table 1:** Seahorse Assay Media (AM)

Reagent	Final concentration	Amount
Seahorse XF DMEM, pH 7.4	N/A	48.5 mL
Seahorse XF 1.0 M glucose solution	10 mM	0.5 mL
Seahorse XF 100 mM pyruvate solution	1 mM	0.5 mL
Seahorse XF 200 mM glutamine solution	2 mM	0.5 mL
**Total**	**N/A**	**50 mL**

***Note:*** Always prepare shortly before use.

33. Wash the cells twice with 100 μl of AM.
34. Add 180 μl of AM. Check for sufficient cell attachment under a microscope (Figure 2A).

**Figure 2:**
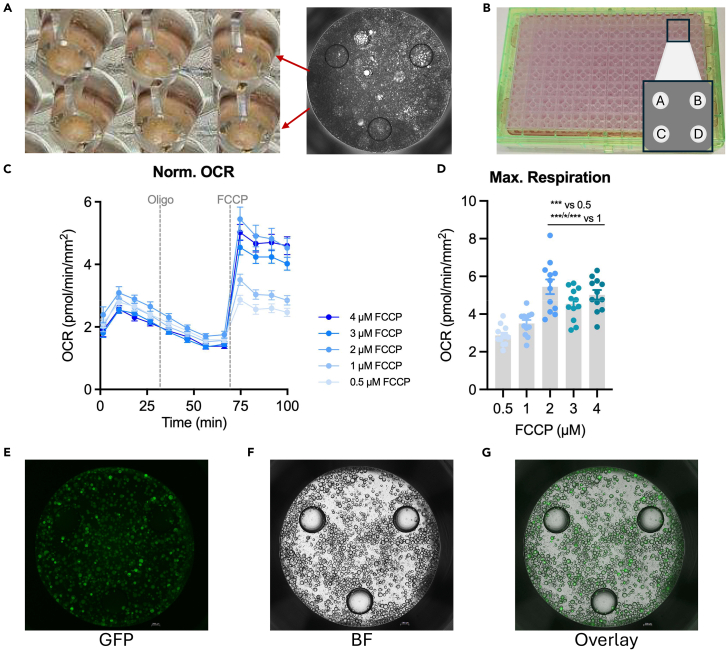
Embedding of mature adipocytes into Matrigel and normalization of OCR measurements to AdipoRed-staining (A) 2× magnification of mature adipocytes embedded in an XFPro-96-well plate using Matrigel two days prior assay. (B) Seahorse sensor cartridge on top of a Hydrobooster (pink) and utility plate (clear) with injection ports A–D. (C) The FCCP concentration elicitating maximal respiration was determined in a titration experiment (0.5 μM - 4 μM). (D) revealing a concentration of 2 μM FCCP sufficient to induce maximal respiration. Data are presented as mean ± SEM. Statistical significance is indicated by asterisks (∗p < 0.05, ∗∗∗p < 0.001) and was determined by one-way ANOVA with Tukey’s post test. (E–G) Mature adipocytes were stained with the AdipoRed reagent immediately after the assay was finished. Representative pictures of stained adipocytes are shown; (E) GFP-channel, (F) bright field, and (G) overlay.
35. Incubate the plate at 37°C in a CO_2_ -free incubator for 1 h.
36. Prepare the stock solutions of oligomycin, FCCP, and rotenone/antimycin A according to Table 2.

**Table 2:** Preparation of the mitochondrial stress test stock solutions

Reagent	Final concentration	Amount of AM to add
Oligomycin stock solution	100 μM	630 μl
FCCP stock solution	100 μM	720 μl
Rot/AA stock solution	50 μM	540 μl
37. Dilute the stock solutions by adding the appropriate volume of AM according to Table 3.

**Table 3:** Preparation of the mitochondrial stress test injections optimized for human mature adipocytes

Reagent	Final concentration	Amount of stock solution	Amount of AM to add
Oligomycin	20 μM	500 μl	2000 μl
FCCP	20 μM	600 μl	2400 μl
Rot/AA	5 μM	300 μl	2700 μl
38. Remove the sensor cartridge from the incubator and load the ports (Figure 2B) with the dilutions prepared in Step 39 with the according volumes given in Table 3.

**Table 4:** Final concentrations and volumes loaded to port A-C for injections

Reagent	Final concentration	Port	Volume per port
Oligomycin	2 μM	A	20 μl
FCCP	2 μM	B	22 μl
Rot/AA	0.5 μM	C	25 μl

***Note:*** During the assay, the volumes in the ports are subsequently added to the wells and diluted to the intended final concentration, as shown in Table 4.

***Note:*** The optimal FCCP concentration may vary depending on the biological material and experimental conditions. In our hands, a titration experiment (0.5 μM – 4 μM FCCP) using freshly isolated human mature adipocytes showed that 2 μM reliably induced maximal respiration without evidence of respiratory suppression at higher concentrations (Figures 2C and 2D). However, due to expected inter-donor variability in primary human adipocytes, users may consider performing an FCCP titration to determine the optimal concentration for their specific samples and experimental setup.

39. Start the Wave Pro program and set up the mitochondrial stress test (MST) assay protocol as stated in Table 5.
  a. Ensure that the order of the injections are set up correctly.
  b. Place the sensor cartridge on the plate holder, making sure to remove the lid and the pink Hydrobooster plate.
  c. Start the calibration.

**Table 5:** Settings for the mitochondrial stress test

Measurement	Cycles	Mix [min]	Wait [min]	Measure [min]
Basal respiration	4	2	2:30	3
Oligomycin (A)	4	2	4	3
FCCP (C)	4	2	2:30	3
Rot/AA (D)	4	2	2:30	3
40. After the calibration step, remove the calibration plate and load the plate with the attached cells.
  a. Load the plate with the correct orientation (A to A).
  b. Remove the lid.
  c. Start the measurement.

***Note:*** To ensure a robust evaluation of the data, a basal OCR of at least 13 pmol/min (i.e., in the third basal measurement cycle) should be achieved after background correction when using the Seahorse XF Pro. Otherwise, evaluation will be difficult, especially after the injection of oligomycin, because the background noise will be too high.

### Assay normalization

**Timing: Immediately after the assay**

Here, we describe an assay normalization method based on AdipoRed™ lipid staining that is specifically tailored for mature adipocytes.

41. After the assay, add 2 μl of the AdipoRed™ reagent per well, and incubate for 15 min at 20–23°C.
42. Perform a plate scan with a 2× objective using a BX-Z800 Microscope (or comparable) using the GFP and bright field channels and making sure the whole well area is detected (Figures 2E–2G).

***Note:*** Some cells may detach during the assay. These cells will not contribute to the OCR because they are floating on the surface. We do not recommend normalizing to total DNA or protein content because this would also involve the floating cells. Instead, we recommend using the AdipoRed lipid staining and normalizing to the area covered by cells, since only these cells contribute to the measurement. If no cells detach, or if there are enough technical replicates per condition, it is also possible to proceed without normalization because the same volumes of the cell-Matrigel suspension were added to each well.

43. Execute a pixel based classification using the open-source software Ilastik.^4^
  a. Use a subset of GFP images to train a classifier that differentiates between cells and background.
  b. Use a subset of the brightfield images and train a classifier that distinguishes between wells and the background.
  c. Analyze your entire image set with these classifiers to obtain probability maps for a) cells and b) wells.

***Note:*** If your images are of high quality, this step might not be necessary. If your images were not evenly illuminated and contain some background noise, you can use a pixel-based classification to eliminate the noise. Subsequently, the well classifier will be used to mask the part of the image that does not contain important information, thereby speeding up the analysis process.

44. Perform an automated image analysis using CellProfiler™.^5^

***Note:*** The area of the well covered by cells is measured in mm^2^. This value is used to normalize the OCR data. For detailed information about the analysis process, refer to the CellProfiler™ pipeline in the supplementary information.

**CRITICAL:** Carefully evaluate whether the CellProfiler™ pipeline settings match your needs. Depending on your microscope settings (e.g., different objectives), you may need to adjust some parameters. Inspect the overlay pictures generated by CellProfiler™ to ensure that the analysis was performed properly.

### Data analysis

**Timing: Any time after the assay**

This step describes the analysis of the mitochondrial stress tests data.

45. Open your experiment in the Wave desktop software.
46. Normalize your data for the area covered by cells, as calculated in Step 46.
47. Use the Seahorse XF Wave software to detect and exclude possible outliers.
48. Export your data as a Seahorse XF Cell Mito Stress Test Report Generator.

**Figure 3:**
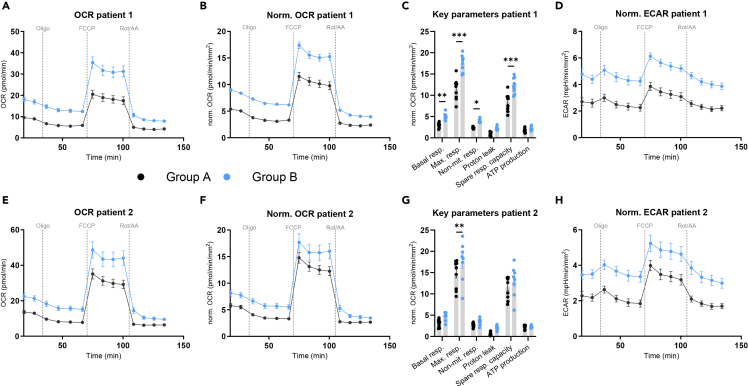
OCR measurements using the Seahorse Analyzer Basal and normalized OCR (A, E and B, F), key mitochondrial parameters (C and G) and normalized ECAR (D and H) of the mitochondrial stress test performed on mature adipocytes from two different patients under two different conditions (control, treatment). Data are presented as mean ± SEM. Statistical significance is indicated by asterisks (∗p < 0.05, ∗∗p < 0.01, ∗∗∗p < 0.001) and was determined by two-way ANOVA with Fisher’s LSD.

***Note:*** Export raw kinetic data to Excel or GraphPad (see examples in Figures 3A, 3B, 3E, and 3F). Analyze the parameters of interest (e.g., as basal respiration, maximal respiration, proton leak, ATP synthesis and spare respiratory capacity) according to the manufacturer’s instructions. Plot the results in a graph (see examples in Figures 3C and 3G).

***Note:*** The extracellular acidification rate (ECAR) is also measured during the MST (Figures 3D and 3H) and may give additional insights into adipocyte metabolism (e.g. glycolytic turnover). However, it is not a direct measurement of glycolysis, rather a summation of acidification produced from various metabolic pathways. A more direct measurement of glycolysis can be achieved using this protocol in combination with the XF Glycolytic Rate Assay.

## Expected outcomes

This protocol enables robust functional assessment of primary human mature adipocytes in a physiologically relevant state. By combining Matrigel-based immobilization with extracellular flux analysis, it allows reproducible characterization of adipocyte bioenergetics following isolation and culture. The approach provides a reliable platform to investigate metabolic alterations associated with environmental stimuli, disease states, or experimental perturbations in human adipocytes.

## Limitations

Mature adipocytes isolated from white adipose tissue depots exhibit inherently low metabolic activity, primarily due to their relatively low mitochondrial content compared to other cell types. This characteristic should be carefully considered during experimental design. Accordingly, this model is not ideal for studies aiming to further reduce oxygen consumption rates (OCR), e.g., by gene knockdown approaches or pharmacological inhibition.

In contrast, this model is particularly advantageous for studies focused on enhancing the metabolic activity of mature adipocytes through genetic manipulation or targeted stimulation. Another application is the comparative analysis of bioenergetic parameters in mature adipocytes derived from metabolically healthy versus metabolically impaired individuals. However, due to interindividual biological variability, certain adipocyte populations may not respond to injection procedures and thus remain inaccessible using this technique.

Despite these limitations, few assays currently enable direct assessment of primary mature adipocyte function independent of other cell types present in adipose tissue. Therefore, this model is a valuable experimental tool for elucidating the mechanisms underlying adipocyte function and dysfunction. Another limitation is the need for enough human adipose tissue to use this cell culture model.

## Troubleshooting

### Problem 1

Cells gather at the edges of the well (Step 27).

### Potential solution

• Make sure you tap the plate after adding each condition to the wells; otherwise, the cell suspension added first will already have polymerized and will no longer be distributed throughout the well.

OR

• Alternatively, vary the volume of cell suspension added per well to reach full well coverage.

### Problem 2

The Matrigel layer is not polymerizing (Step 28).

### Potential solution

• Make sure that all the free lipid on top of the adipocytes is removed; otherwise, Matrigel will not polymerize properly. Another option is to adjust the Matrigel-to-cell suspension ratio to 2:1 to guarantee polymerization. It is also recommended to incubate the Seahorse microplate for 16–24 h at 37°C and place the plate on top of a flask filled with pre-warmed water.

### Problem 3

Low basal OCR (Step 48).

### Potential solution

• There may be too few cells in the well, or cell viability may be compromised. Add more Matrigel-cell-suspension per well or try an adjusted dilution ratio. Prolonged cold exposure or mechanical stress can also compromise cell viability and fitness. Make sure the cells are kept on ice for a maximum of 1 min and use wide-bore pipette tips for this cell type.

OR

• For some patients, the basal cell respiration will be too low without any treatment to overcome the required basal respiration (13pmol/min) regardless of cell number. If basal respiration does not reach 13 pmol/min, the quality of the measurement cannot be guaranteed. Since these cells are derived from actual patients, there will always be significant biological variance, especially due to differences in age, sex, and body weight. However, if you observe the typical curve after injections, as shown for patient 1, the data may still be useful, as the treatment of group B did lead to significant changes in OCR.

### Problem 4

Mature adipocytes do not respond to the injections (Step 48).

### Potential solution

• It is crucial to ensure that inhibitor stocks are prepared correctly and are fresh. Replace them with new, fresh stocks when the adipocytes’ response to the inhibitors decreases over time.

OR

• Some patient samples may be non-responders.

## Resource availability

### Lead contact

Requests for further information, resources, and reagents should be directed to and will be fulfilled by the lead contact, John T. Heiker (john.heiker@helmholtz-munich.de).

### Technical contact

Technical questions on executing this protocol should be directed to the technical contact, Claudia Gebhardt (claudia.gebhardt@helmholtz-munich.de).

### Materials availability

This study did not generate new unique reagents.

### Data and code availability

The CellProfiler pipeline is provided as a supplementary file to this article.

## Acknowledgments

This work was supported by grants from the 10.13039/501100001659Deutsche Forschungsgemeinschaft, project no. 209933838-SFB1052 (obesity mechanisms: C7 to J.T.H.). H.B. is supported by a doctoral scholarship from the 10.13039/501100004350Studienstiftung des Deutschen Volkes. We thank Prof. Dr. Stefan Langer and Dr. Rima Nuwayhid from the Division of Plastic, Aesthetic and Special Hand Surgery at University Hospital Leipzig for providing adipose tissue samples. We are grateful to all donors of adipose tissue samples. The graphical abstract was created with BioRender (https://BioRender.com/sg0ws3e).

## Author contributions

All authors conceptualized, initiated, and developed the project. H.B. and C.G. performed the experiments and analyzed the data. H.B. and J.T.H. wrote the manuscript with input from C.G. J.T.H. supervised the project.

## Declaration of interests

The authors declare no competing interests.

## Supplemental information

Data S1. CellProfiler pipeline for automated image analysis, related to step 44
