# Supplementary figures and images for: Protocol for measuring mitochondrial respiration in mature human adipocytes using the Seahorse XF analyzer
Source: STAR Protoc. 2026 Apr 25;7(2):104520. doi: 10.1016/j.xpro.2026.104520 (PMC13134015; doi:10.1016/j.xpro.2026.104520)

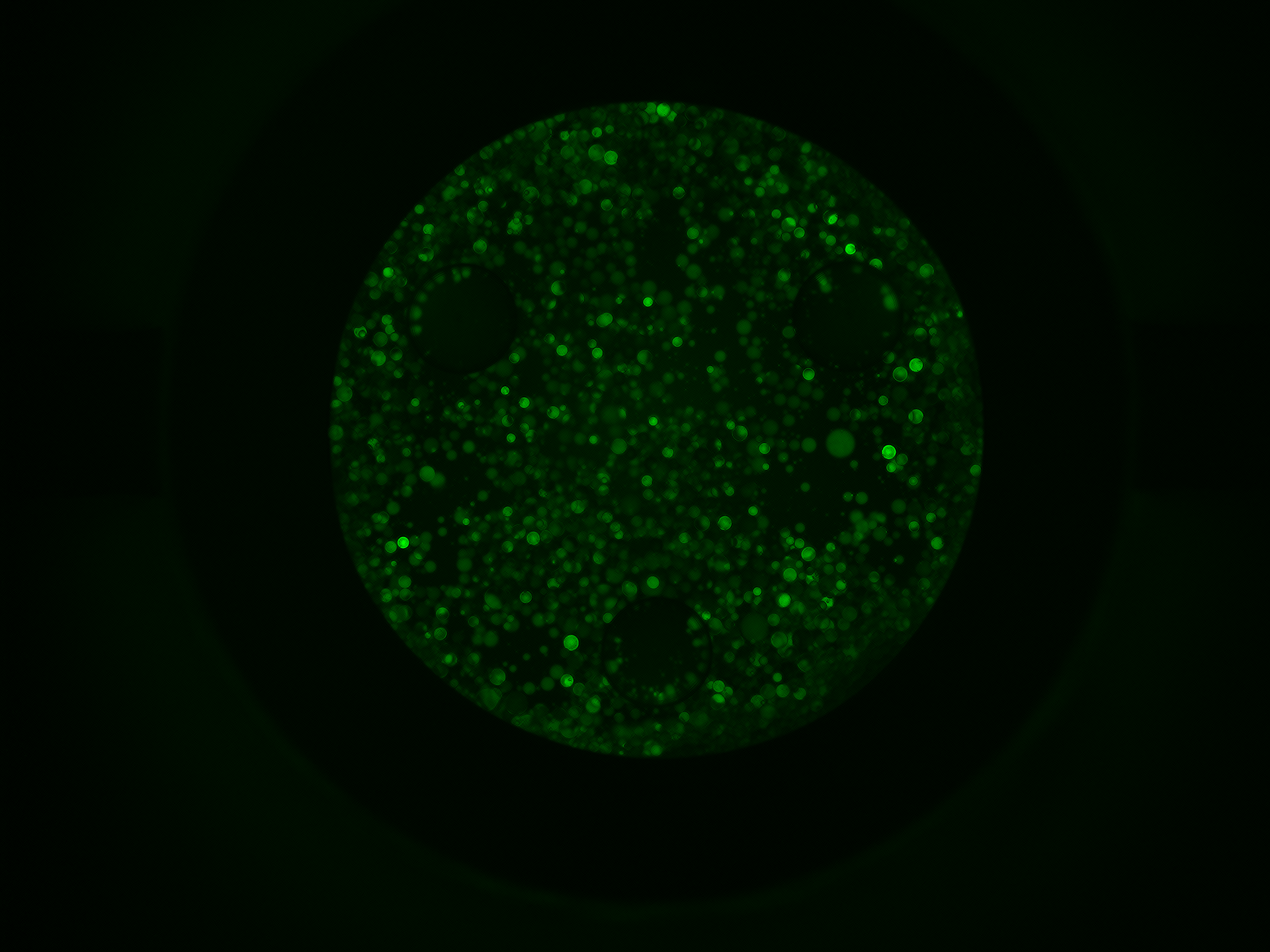

Supplement: Data S1. CellProfiler pipeline for automated image analysis, related to step 44 [file mmc1.zip › CellProfiler Pipeline/Example pictures/Adipored.tif]

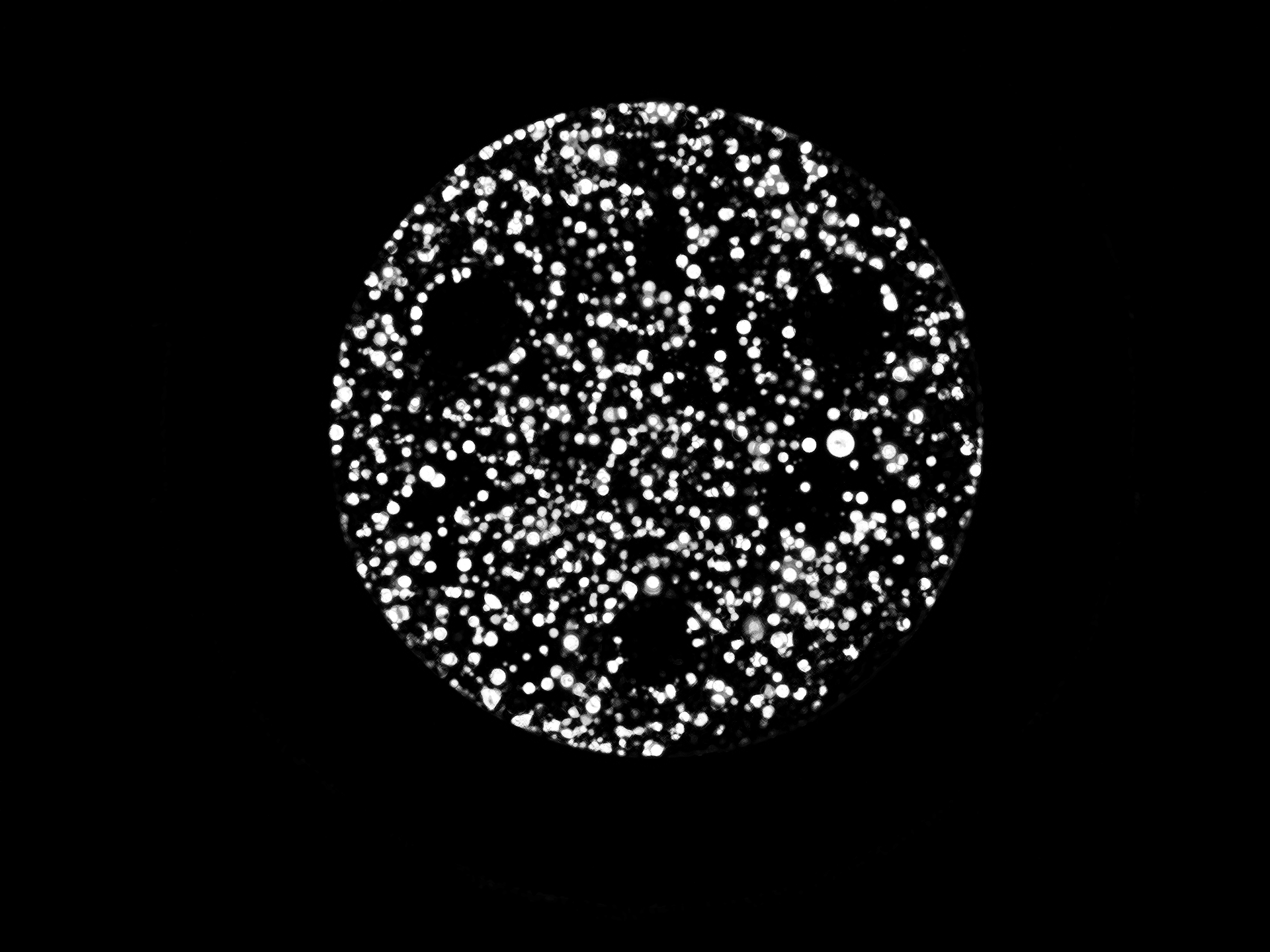

Supplement: Data S1. CellProfiler pipeline for automated image analysis, related to step 44 [file mmc1.zip › CellProfiler Pipeline/Example pictures/Probabilities_cell.png]

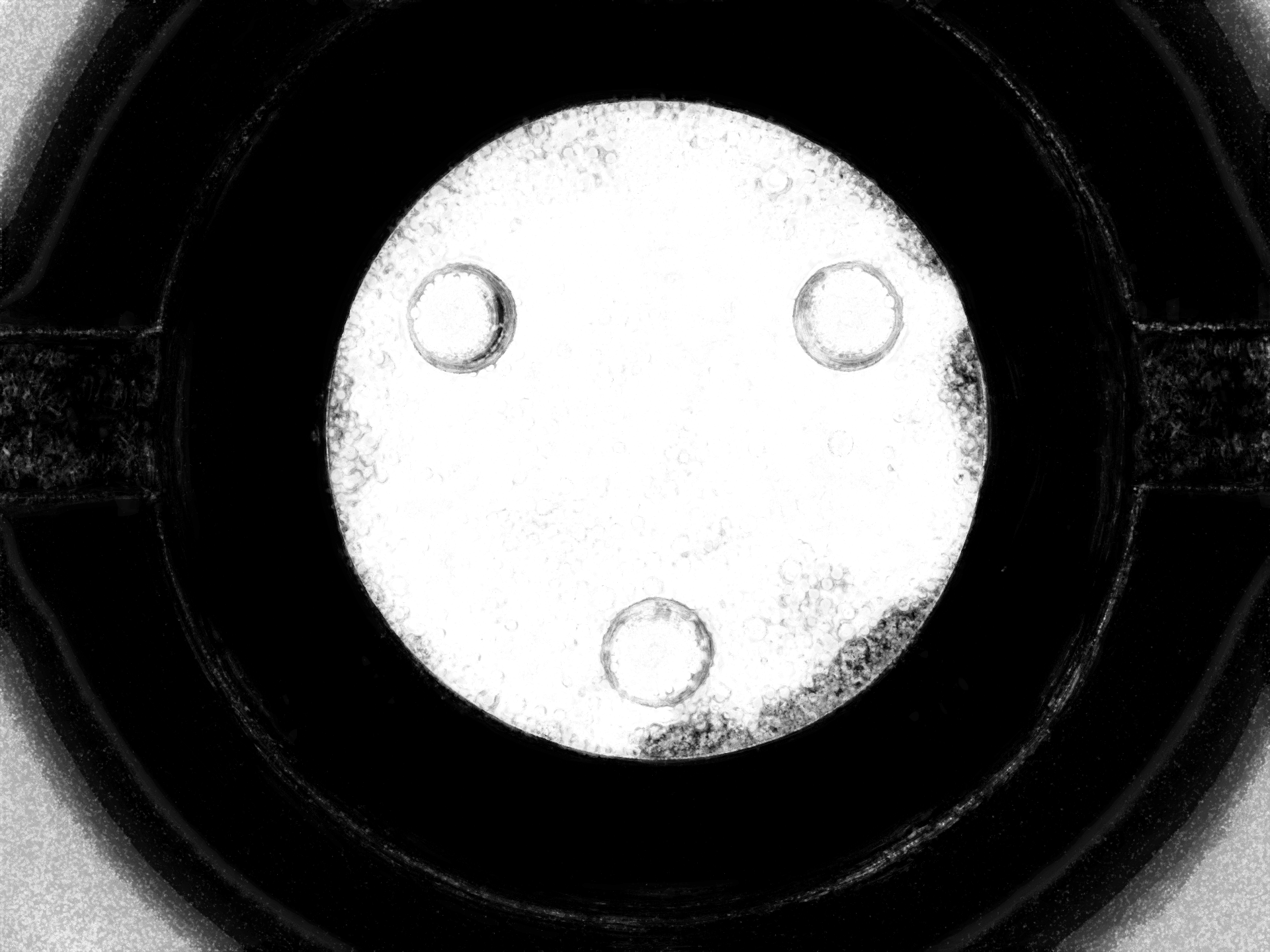

Supplement: Data S1. CellProfiler pipeline for automated image analysis, related to step 44 [file mmc1.zip › CellProfiler Pipeline/Example pictures/Probabilities_well.png]
